# Imaging in Septic Arthritis With Inflammatory Neuritis: Diagnosing a Rare Complication

**DOI:** 10.7759/cureus.61771

**Published:** 2024-06-05

**Authors:** Tushar Kalekar, Apurvaa Pachva, Sai Pavan Kumar

**Affiliations:** 1 Department of Radiology, Dr. D. Y. Patil Medical College, Hospital and Research Centre, Dr. D. Y. Patil Vidyapeeth, Pune (Deemed to Be University), Pune, IND

**Keywords:** s: intramedullary nail, septic arthritis of hip, pain of hip, femur and fracture, s: magnetic resonance imaging

## Abstract

After femoral neck fractures, hip septic arthritis and inflammatory neuritis are extremely rare. For the purpose of making an accurate diagnosis and preventing serious joint damage, early clinical examination and imaging are crucial. Very few studies have thoroughly described the intraoperative and radiographic results of these disorders. We present the case of a 36-year-old man who developed right hip pain one month after undergoing surgery for a right femur head fracture. Magnetic resonance imaging and biopsy revealed the presence of septic arthritis and inflammatory neuritis. Following the initiation of conservative treatment, the patient is receiving routine follow-up. This case highlights its distinctive features and challenges of diagnosing this entity, emphasizing the significance of vigilant clinical evaluation and imaging modalities for prompt management and the best possible outcomes for patients.

## Introduction

Bacteria, but occasionally fungal, mycobacterial, viral, or other uncommon pathogens, cause septic arthritis, which is inflammation of the joints [1-3]. The extension of inflammation along the nerve roots, which can manifest as lower limb numbness, tingling, or even a foot drop, can further complicate matters. Post-surgical inflammatory neuritis, unlike other forms of neuritis or neuropathy, doesn't respond well to steroids [1]. Septic arthritis and inflammatory neuritis, especially post-operatively, are rare orthopedic emergencies that can damage joints and increase morbidity and mortality.

A plain radiograph may show expanded joint gaps, soft tissue bulging, or subchondral bone alterations (late discovery), but it remains non-specific [4]. Magnetic resonance imaging (MRI) can detect early joint effusion, abnormalities in surrounding soft tissue and bone, and cartilaginous involvement.

Septic arthritis and inflammatory hip neuritis following a femoral neck fracture are extremely rare complications. As a result, identifying this entity in a timely manner with clinical examination and imaging confirmation is critical to preventing extensive articular damage. There is a paucity of research detailing the radiological findings of septic arthritis and post-operative inflammatory neuritis, as well as the subsequent intra-operative observations.

## Case presentation

This is a 36-year-old male patient who has had a traumatic fracture of the right femur head with posterior displacement. He underwent open reduction and internal fixation with Herbert screw, with no hypertension, diabetes mellitus, or steroid use. He presented to the Department of Orthopedics with complaints of pain in the right hip for one month after surgery, which was insidious in onset, gradually progressive, aggravated when walking, and partially relieved when resting. This was accompanied by pain in the right thigh and calf, as well as lower limb numbness and tingling. However, there was no history of swelling, fever, or recent falling. There was no immediate postoperative wound discharge or implant failure.

Upon examination, the patient had tachycardia. The electrocardiogram (ECG) is normal, the erythrocyte sedimentation rate (ESR) is elevated by 24 mm/hour, the C-reactive protein (CRP) is elevated by 2.09, and the complete blood picture (CBP), blood sugar level (BSL), and other lab parameters are within normal limits. He was afebrile at the time of the examination. The local examination of the right thigh revealed an antalgic gait, with the limb in an adducted and internally rotated position. The previous surgical scar appears to have healed with primary intent. We found the rest of the systemic examination to be normal.

**Figure 1:**
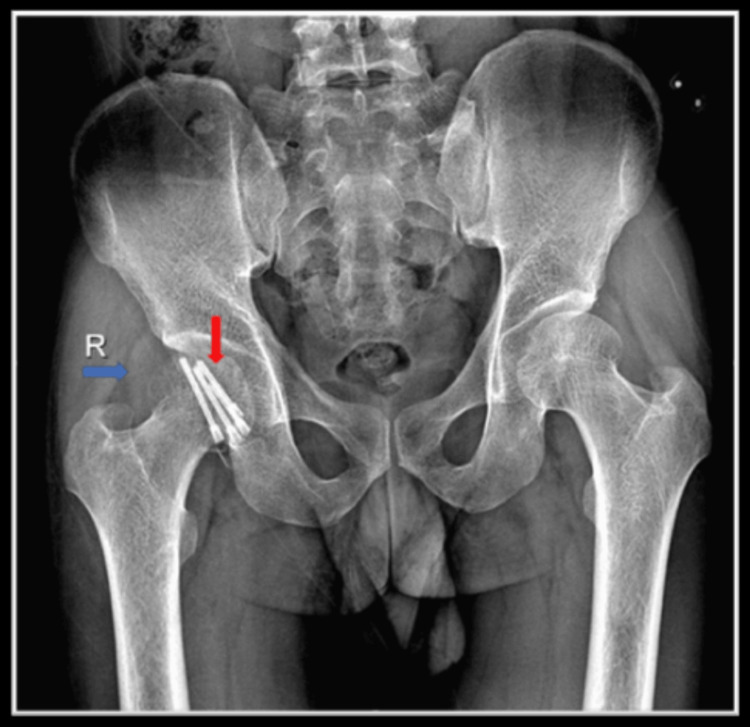
Postoperative plain radiograph of pelvis anteroposterior view showing Herbert screws (shown by red arrow) and the elevation of fat plane (shown by blue arrow)

To determine the cause of the pain, we performed a right hip radiograph, which revealed signs of union, a widening of joint spaces, and elevated fat planes (Figure 1).

We obtained multiplanar, multi-echo sequences for the lumbosacral spine with lumbar plexus to further delineate the cause on Siemens MAGNETOM Vida 3 Tesla MRI (Siemens Healthineers, Erlangen, Germany). The protocol utilized used such as T1 sagittal, T2 sagittal, coronal T1, coronal short tau inversion recovery (STIR), STIR sagittal T2, diffusion-weighted image, sequences, and multiplanar reformation (MPR). To delineate the cause further, multiplanar multi-echo sequences for the lumbar plexus were taken on Siemens 3 Tesla MRI. The protocol included coronal T1, transverse T1 and T2, coronal STIR, transverse STIR T2, and diffusion-weighted images (Table 1).

**Table 1:** MRI pulse sequence parameter protocol for imaging of lumbosacral spine with lumbar plexus MRI: magnetic resonance imaging; TE: echo time, TR: repetition time; FOV: field of view; STIR: short tau inversion recovery; DWI: diffusion-weighted image

MRI pulse sequence parameter protocol	Coronal T1	Coronal STIR	Transverse STIR T2	Transverse T1	Transverse T2	DWI (50, 800)
TE (milliseconds)	9.1	29	38	10	100	59
TR (milliseconds)	650	6040	4530	800	5080	2600
Slice thickness (millimeters)	3.0	3.0	3	3	3	3.5
FOV (millimeters)	300	380	320	300	300	350

**Figure 2:**
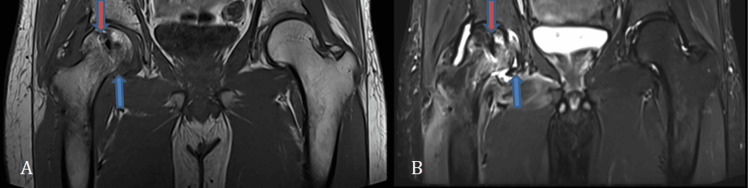
A) Coronal T1 weighted image; B) Coronal T2 STIR image STIR: short tau inversion recovery These images show susceptibility artifacts noted in the right femoral head due to internal fixation with screws (shown by red arrow). Altered signal intensity is noted in the right femoral head and neck region, appearing hypointense on T1 and hyperintense on T2 and STIR sequences, which suggests marrow edema with cortical irregularities and erosions

On all MRI sequences, the right femoral head exhibits susceptibility artifacts as a result of internal screw fixation. Altered signal intensity is noted in the right femoral head and neck region, appearing hypointense on T1, hyperintense on T2, and STIR, indicating marrow edema with cortical irregularities and erosions (Figures 2A-2B).

**Figure 3:**
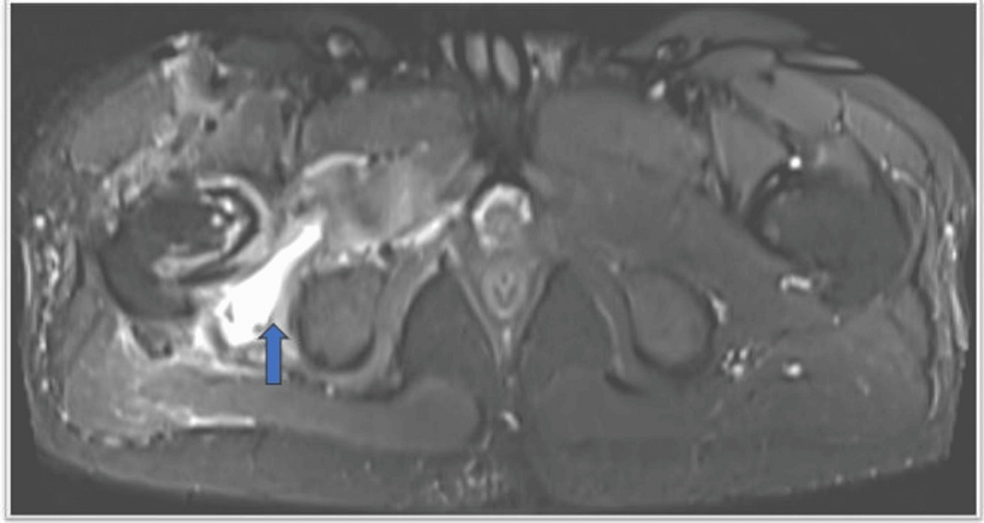
STIR hyperintense signal noted in the surrounding soft tissue with localized fluid collection around the femoral neck (shown by blue arrow) STIR: short tau inversion recovery

The soft tissue around the femoral neck also had a T2/STIR hyperintense signal, and there was a collection of fluid around the femur's neck (Figure 3).

**Figure 4:**
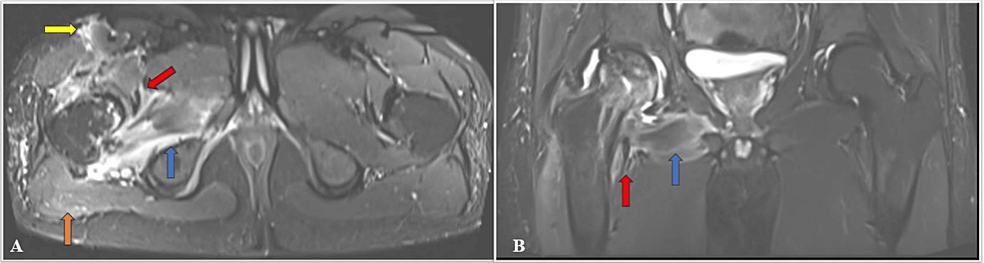
A) T2 STIR axial image; B) T2 STIR coronal image STIR: short tau inversion recovery These images show STIR hyperintense signal is noted in the obturator externus muscle (shown by blue arrow), iliopsoas (shown by red arrow), rectus femoris (shown by yellow arrow), and gluteus muscle (shown by orange arrow)

The STIR hyperintensity signal is noted in the obturator externus muscle, iliopsoas, and rectus femoris. Intramuscular and subcutaneous edema is noted around the femoral head and neck region, with a fluid collection of an approximate volume of 10 cc, tracking up to the superficial subcutaneous plane and posteriorly along the pyriformis, gluteus minimus, and adductor magnus muscles (Figures 4A-4B).

**Figure 5:**
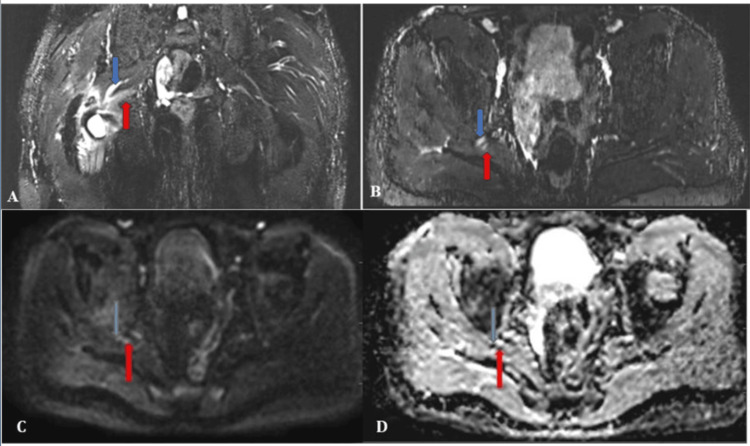
A) Coronal MIP image; B) Axial MIP image; C) DWI; D) ADC MIP: maximum intensity projection image; DWI: diffusion-weighted imaging; ADC: apparent diffusion coefficient Hyperintensity and increased thickness are noted in the right sciatic nerve (shown by blue arrow) and right posterior cutaneous nerve (shown by red arrow) Figures (C) and (D) show diffusion restriction with a low ADC value

This is causing a mass effect and extension along the sciatic nerve and posterior cutaneous nerve of the thigh, which are edematous and swollen and show a STIR hyperintense signal (Figures 5A-5D).

Findings related to the lumbosacral spine and lumbar plexus include: mild edema is observed in the right S1 and L5 exiting nerve roots near the greater sciatic foramina. Visualized vertebral body heights are maintained with normal marrow signal intensity. The lower cord and conus appear normal. The lumbosacral plexus appears normal in size, signal, and course. The left sciatic nerve, femoral nerve, obturator nerve, and lateral femoral cutaneous nerves also appear normal in size, signal, and course.

**Figure 6:**
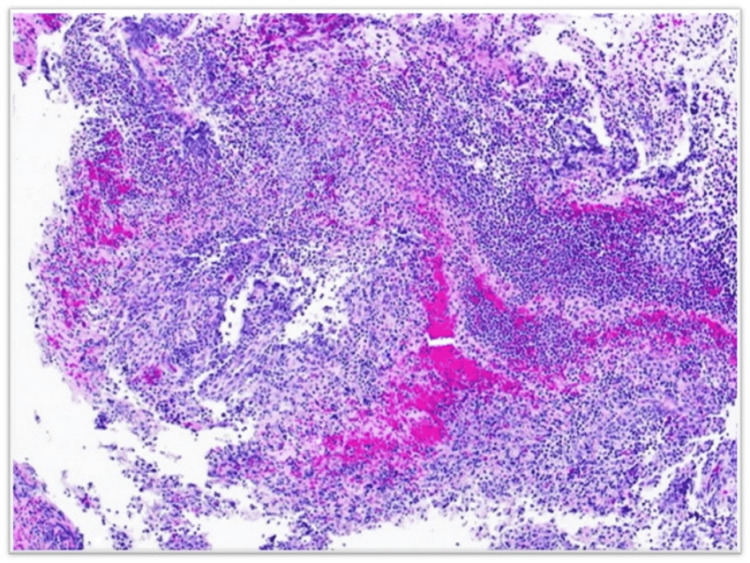
Histopathology image showing fragmented fibro collagenous tissue with neutrophils, few lymphocytes, and eosinophils

After the MRI, a working diagnosis of an infective pathology in the right femur and likely a septic arthritis abscess with inflammatory neuritis. A tru-cut biopsy of synovial articular cartilage revealed fragmented fibro-collagenous tissue with a necroinflammatory reaction that included neutrophils, a few lymphocytes, and eosinophils, confirming the radiological diagnosis of septic arthritis (Figure 6).

Following the confirmation of the diagnosis by histopathology, we initiated the patient on IV antibiotics based on culture and sensitivity. These included an injection of piperacillin and tazobactam 4.5 gm thrice a day, as well as an injection of metronidazole 100 mL IV twice a day. Additionally, we administered low-dose oral prednisolone 20 mg twice a day, which we tapered over the next four weeks. We immobilized the affected limb to alleviate the pain and administered IV analgesics (injection diclofenac), along with oral ketorolac. The patient experienced a single fever episode on the third day of treatment, prompting the evaluation of blood and urine cultures, which showed no growth. The patient demonstrated a significant reduction in pain, no further fever spikes, and an improvement in numbness.

Following 10 days of IV antibiotics, the doctor discharged the patient and recommended using a walker for partial weight bearing. We recommended limited isometric exercises as part of his physiotherapy, along with ultrasound and infrared therapy for the affected hip. The orthopedic OPD reviewed the patient, and a clinical examination revealed an improvement in antalgic gait.

## Discussion

There are only three known reports of septic arthritis following femoral neck fractures, which indicates how uncommon this complication is. Hearth et al. reported two cases, while Colak et al. recorded three, and Chewakidakarn et al. reported one. The age range of the patients in all publications, ranging from 48 to 96 years old, was associated with immunocompromised hosts [3-5]. The 36-year-old man we treated had no co-morbidities and developed septic arthritis four weeks after the index surgery. This is much later than the other cases reported so far. Similar to our case, which developed septic arthritis about two weeks after the fracture, the majority of the cases in the prior publications underwent definitive surgery at least two weeks after the initial injury [3-6].

The infection can originate from any system in the body, making it difficult to diagnose and treat septic arthritis. Radiological findings, however, are characteristic and can help guide not only the treatment but also the efficacy of antibiotics or the adequacy of surgical debridement. Plain radiographs can show features such as fat plane loss, cortex elevation, malunion, or an increase in joint space. MRI is specifically used to identify soft tissue changes caused by septic arthritis.

Lee et al. [6] found that six of the nine people diagnosed with septic arthritis showed changes in bone marrow signal intensity. Kwack et al. [7] observed changes in the amplitude of the signal in three out of nine patients diagnosed with the condition, despite the fact that the difference in bone marrow signal intensity between the two groups in our experiment was not highly significant (p = 0.074). Neither the study by Kwack et al. nor the trial by Lee et al. any statistically significant differences between the two groups in the number of people with abnormal soft-tissue signal intensity, the amount of effusion, or synovial hypertrophy in the hip that was under study. This pattern was evident in both of the studies mentioned above. After surgery, inflammatory neuritis can be difficult to treat because it does not respond to the same treatments as other inflammatory nerve illnesses that are equivalent.

Additionally, we diagnosed our patient with a rare combination of septic arthritis and inflammatory neuritis. Laughlin et al. reported that seven individuals with a presentation similar to ours experienced weakness in their legs 30 days after hip replacement surgery. This was due to microvasculitis and the involvement of the lumbosacral plexus on neural biopsy [8]. Additionally, these individuals had a similar description to ours. An identical group had previously published their experience in a tertiary center, where they identified 33 patients with postsurgical inflammatory neuropathies. This experience was based on clinical characteristics, a nerve biopsy, electromyography, and MRI.

## Conclusions

Specific clinical features limit the occurrence of septic arthritis with inflammatory neuritis following a femur fracture and surgical intervention. Timely radiological investigations can be useful to make an appropriate diagnosis and prevent the spread of infection, implant failure, and neurological complications such as paralysis and lumbar plexus neuropathy.
